# Attitudes of medical workers in China toward artificial intelligence in ophthalmology: a comparative survey

**DOI:** 10.1186/s12913-021-07044-5

**Published:** 2021-10-09

**Authors:** Bo Zheng, Mao-nian Wu, Shao-jun Zhu, Hong-xia Zhou, Xiu-lan Hao, Fang-qin Fei, Yun Jia, Jian Wu, Wei-hua Yang, Xue-ping Pan

**Affiliations:** 1 School of Information Engineering, Huzhou University, Zhejiang 313000 Huzhou, China; 2 Zhejiang Province Key Laboratory of Smart Management & Application of Modern Agricultural Resources, Huzhou University, 313000 Huzhou, China Zhejiang Province; 3 College of Computer and Information, Hehai University, 210013 Nanjing, China Jiangsu; 4 Department of Endocrinology, First Affiliated Hospital of Huzhou University, 313000 Huzhou, China Zhejiang; 5 School of Medicine, Huzhou University, 313000 Huzhou, China Zhejiang; 6 Zhejiang University Real Doctor AI Research Center, 310000 Hangzhou, Zhejiang P.R. China; 7 Affiliated Eye Hospital of Nanjing Medical University, No.138 Hanzhong Road, Gulou District, 210029 Nanjing, Jiangsu China; 8 First People’s Hospital of Huzhou, 313000 Huzhou, China Zhejiang

**Keywords:** Artificial intelligence, Ophthalmic artificial intelligence, Artificial intelligence in ophthalmology, Artificial intelligence in medicine, Replacing doctors, Doctor replacement, Medical ethics

## Abstract

**Background:**

In the development of artificial intelligence in ophthalmology, the ophthalmic AI-related recognition issues are prominent, but there is a lack of research into people’s familiarity with and their attitudes toward ophthalmic AI. This survey aims to assess medical workers’ and other professional technicians’ familiarity with, attitudes toward, and concerns about AI in ophthalmology.

**Methods:**

This is a cross-sectional study design study. An electronic questionnaire was designed through the app Questionnaire Star, and was sent to respondents through WeChat, China’s version of Facebook or WhatsApp. The participation was voluntary and anonymous. The questionnaire consisted of four parts, namely the respondents’ background, their basic understanding of AI, their attitudes toward AI, and their concerns about AI. A total of 562 respondents were counted, with 562 valid questionnaires returned. The results of the questionnaires are displayed in an Excel 2003 form.

**Results:**

There were 291 medical workers and 271 other professional technicians completed the questionnaire. About 1/3 of the respondents understood AI and ophthalmic AI. The percentages of people who understood ophthalmic AI among medical workers and other professional technicians were about 42.6 % and 15.6 %, respectively. About 66.0 % of the respondents thought that AI in ophthalmology would partly replace doctors, about 59.07 % having a relatively high acceptance level of ophthalmic AI. Meanwhile, among those with AI in ophthalmology application experiences (30.6 %), above 70 % of respondents held a full acceptance attitude toward AI in ophthalmology. The respondents expressed medical ethics concerns about AI in ophthalmology. And among the respondents who understood AI in ophthalmology, almost all the people said that there was a need to increase the study of medical ethics issues in the ophthalmic AI field.

**Conclusions:**

The survey results revealed that the medical workers had a higher understanding level of AI in ophthalmology than other professional technicians, making it necessary to popularize ophthalmic AI education among other professional technicians. Most of the respondents did not have any experience in ophthalmic AI but generally had a relatively high acceptance level of AI in ophthalmology, and there was a need to strengthen research into medical ethics issues.

## Background

In recent years, with the increase of computational speed, neural networks have regained prosperity after hitting its rock bottom. The deep convolutional neural network AlexNet [1] winning the first place in the ImageNet competition has led to the rapid development of deep learning technology. After that, deep learning network models [2–10] have emerged generation after generation, which has accelerated the development of artificial intelligence (AI) technology. Through AI, researchers can make the preliminary diagnosis of skin cancers, achieve rapid intraoperative diagnosis of brain tumors, diagnose 55 common diseases in pediatrics based on electronic medical records in Chinese, identify rare genetic diseases through facial photographs, and generate the findings that early and frequent patient movements can reduce the risk of post-intensive care syndrome and long-term dysfunction after analyzing patients’ movement activities in the intensive care units. [11–15]

Medical instruments are frequently used in clinical examinations in modern medicine, and imaging equipment is an important part. Imaging equipment is used for X-ray imaging, magnetic resonance imaging, ultrasound imaging, tomography imaging, and fundus photography, etc. The data in the ophthalmology field is diverse and huge, among which the most common types are fundus photographs and optical coherence tomography (OCT) images, making it the most extensively researched field with AI. Fundus photography and OCT are regular examinations used in ophthalmology, through which a vast amount of high-quality standard images can be obtained. These images are suitable for analysis and process by AI deep learning technology to further assist doctors in diagnosing ophthalmopathies. Using deep learning technology in AI, Google researchers have created an algorithm that can detect diabetic retinopathy and macular edema. By analyzing human retinal images, this algorithm can very accurately predict risk factors affecting cardiovascular health. [16, 17] The researchers from Sun Yat-sen University in China have developed a deep learning model called CC-Cruiser for recognizing congenital cataract, which is also able to diagnose blinding diseases such as age-related macular degeneration and diabetic macular edema after trained with deep learning algorithms based on OCT images [18, 19]. Besides, there are related studies that use AI technology for the segmentation of ophthalmic images [20, 21], and the classification of ophthalmopathies [22–24], etc.

Artificial intelligence technology has been studied so extensively in ophthalmology that some even have been on the commercializing stage [25], thus leading to some people believing that AI may be able to replace doctors. Some researchers investigated the attitudes of medical undergraduates to the application of AI in radiology and medicine [26], the attitudes of medical students in other regions to AI [27], the influence of artificial intelligence on radiology [28], as well as how to cope with the ethical challenges in medical AI [29, 30], etc. However, few people know about medical workers’ familiarity with and their attitudes toward AI in ophthalmology. For this purpose, a questionnaire was designed to assess medical workers’ (health care workers or medical students) understanding level of and their attitudes toward AI. Meanwhile, the questionnaire also surveyed other professional technicians (engineers, teachers, technicians, experimenters, etc. in non-medical fields) using the same questions as a comparison.

The survey of AI in ophthalmology-related questions cannot only evaluate the attitudes of medical workers (health care workers or medical students) and other professional technicians toward AI and clarify the dilemma facing the current technological development; it can also provide theoretical guidance for its future practice and application. At present, in the development of AI in ophthalmology, there are a lot of existing and foreseeable medical ethical problems. For example, the medical responsibilities are unclear, the problem of the security of patient privacy data and the risk of the weakening of the doctor’s professional status, etc. Analysis of the causes of these problems through this survey may help to put forward corresponding countermeasures so that we can draw on advantages and avoid disadvantages in the future development of AI in ophthalmology.

## Methods

Using the Questionnaire Star APP (a professional questionnaire survey app in China, easy to edit and distribute survey questionnaires), we designed an electronic questionnaire that consisted of four parts. The first part was the respondent’s basic information, including the respondent’s sex, age, educational level, place of residence, work area and professional title; the second part was about the respondent’s basic understanding of AI, including whether the respondent understood AI, medical AI, and AI in ophthalmology, as well as the respondent’s evaluation of the current development of AI in ophthalmology; the third part was about the respondent’s attitude to AI, including whether he/she thought AI in ophthalmology would replace doctors, whether he/she had experience in AI in ophthalmology application, and his/her acceptance level of AI in ophthalmology; and the fourth part was about the respondent’s concerns about AI, which included the respondent’ specific concerns about AI in ophthalmology and whether he/she thought it was necessary to strengthen medical ethics research in the field.

According to the pre-investigation experience and the principle of the rank order scale, the basis for the classification of the four ranks is determined. There were 4 grading used to reflect the basic understanding of AI in the second part of the electronic questionnaire. The grading “Completely” means the understanding level of AI ≥ 90 %; The grading “Almost” means the understanding level of AI between 50 % and 89 % (include 50 % and 89 %); The grading “A little” means the understanding level of AI between 10 % and 49 % (include 10 % and 49 %); The grading “Not understand” means the understanding level of AI ≤ 9 %.

There were 4 grading used to reflect the respondent’s attitude to AI in the third part of the electronic questionnaire. The grading “Completely” means the acceptance level of ophthalmic AI ≥ 80 %; The grading “Partly” means the acceptance level of ophthalmic AI between 20 % and 79 % (include 20 % and 79 %); The grading “No” means the acceptance level of ophthalmic AI ≤ 19 %; The grading “Don’t understand” means the respondents didn’t understand the question.

Respondents of the questionnaire were mainly members of the Zhejiang Society of Mathematical Medicine, with their locations covering various cities and counties mainly in Zhejiang Province. They worked as ophthalmologists, medical students, AI technicians, and professional technicians in other fields. Their educational levels were above junior high school and could understand the questionnaire well. The questions in the questionnaire have been investigated, sorted and summarized repeatedly in a broad and deep way. The survey is a targeted group survey. Questionnaires were sent to medical workers through professional ophthalmological or medical intelligence groups and other professionals through related professional groups. Before we collect the questionnaires for statistical analysis, each respondent was invited to fill out the questionnaire once based on a voluntary and anonymous principle and was informed that the results of the survey would be further used for statistical assessment and publication.

This study is an epidemiological survey research design, containing 15 questions, and usually requires a sample size of at least 5–10 times the number of questions, usually about 15 times. Therefore, the sample we selected is between 15 and 20 times the number of questionnaires, with 291 medical workers and 271 other professional technicians. This study mainly uses statistical analysis, chi-square analysis and odds ratio analysis. The relevant indicators include statistical analysis results, chi-square value, odds ratio (OR) value, Probability (P) value and 95 % CI. A total of 562 respondents were counted, with 562 valid questionnaires returned. The results of the questionnaires are displayed in an Excel 2003 form.

According to the Article 3 of the Measures for the Ethical Review of Biomedical Research Involving Humans issued by the National Health and Family Planning Commission in 2016, ethical review is unnecessary for the study.

## Results

### Basic information of respondents

Of the 562 respondents, 291 were medical workers (51.8 %) with the rest being other professional technicians (48.2 %). As shown in Table 1, nearly half of them were from prefecture-level cities (47.7 %), about 1/4 (24.9 %) were from provincial capital cities, and the remaining were from other regions (27.4 %). 10.0 % had a doctor’s degree or higher education, 13.0 % had a master’s degree, and 77.1 % had a bachelor’s degree or lower education; 31.5 % had senior titles, 25.4 % were with intermediate titles, and 32.0 % were primary and ungraded professionals. The complex structure of these respondents had relatively good social representative value.

**Table 1:** Respondents’ basic information (*N* = 562)

Characteristic	Respondents n(%)
**Sex**	
Male	216(38.4)
Female	346(61.6)
**Age (years)**	
25 or less	70(12.5)
25–45	340(60.5)
45 or more	152(27.1)
**Work area**	
Medical worker (health care worker or medical student)	291(51.8)
Other professional technicians	271(48.2)
**Place of residence**	
Provincial capital	140(24.9)
Prefecture-level city	268(47.7)
County and below	152(27.1)
Abroad	2(0.4)
**Education**	
Doctor and above	56(10.0)
Master	73(13.0)
Bachelor	244(43.4)
Other lower education	189(33.6)
**Professional title**	
Ungraded	180(32.0)
Primary	62(11.0)
Intermediate	143(25.4)
Senior	177(31.5)

### Respondents’ basic understanding and attitudes towards AI

In Table 2, the percentage of respondents who completely understood and almost understood AI was 37.9 %, who understood a little was 52.1 %, and who didn’t understand AI was 10.0 %; the percentage of respondents who completely understood and almost understood medical AI was 31.7 %, who understood a little was 44.3 % and who didn’t understand medical AI was 24.0 %; the percentage of respondents who completely understood and almost understood AI in ophthalmology was 29.6 %, who understood a little was 34.5 %, and who didn’t understand AI in ophthalmology was 35.9 %. That is to say, the proportion of respondents whose understanding level of AI, medical AI, and AI in ophthalmology was “completely”, “almost” or “a little” was gradually decreasing in the mentioned order (AI, medical AI, and ophthalmological AI), while the proportion of people who did not know about AI, medical AI, and AI in ophthalmology was gradually increasing in the same order.

**Table 2:** Respondents’ basic understanding of artificial intelligence (*N* = 562)

Understanding level	Respondents n(%)
**Artificial intelligence**	
Completely	39(6.9)
Almost	174(31.0)
A little	293(52.1)
Not understand	56(10.0)
**Medical artificial intelligence**	
Completely	33(5.9)
Almost	145(25.8)
A little	249(44.3)
Not understand	135(24.0)
**Ophthalmic artificial intelligence**	
Completely	34(6.1)
Almost	132(23.5)
A little	194(34.5)
Not understand	202(35.9)
**Current development of ophthalmic artificial intelligence**	
Very good	109(19.4)
Good	240(42.7)
Average	198(35.2)
Poor	15(2.7)

Among the respondents in Table 2, there were 19.4 % of them thought that the current development of AI in ophthalmology was very good. About 42.7 % of respondents thought that its current development was good. There were 35.2 % of respondents thought that its current development was average. As shown in Table 3, on the question of whether AI in ophthalmology would replace doctors, there were 24.0 % of respondents said no. But 66.0 % of respondents thought it would partly replace doctors. There were 69.4 % of the respondents had no ophthalmic AI-related experience while the rest had applied or experienced AI in ophthalmology. There were 59.1 % of the respondents had a relatively high acceptance level of AI in ophthalmology, with only 2.1 % against it.

**Table 3:** Respondents’ attitudes toward artificial intelligence (*N* = 562)

AI in ophthalmology will replace ophthalmologists	Respondents n(%)
Completely	10(1. 8)
Partly	371(66.0)
Not	135(24.0)
Don’t understand	46(8.2)
**Do you have any experience with AI in ophthalmology?**	Respondents n**(%)**
Being using or about to use	29(5.2)
Having applied	49(8.7)
Having experienced	94(16.7)
Having no related experience	390(69.4)
**Acceptance level of AI in ophthalmology**	Respondents n**(%)**
Completely	332(59.1)
Partly	218(38.8)
Not accept	10(1.8)
Strongly resist	2(0.4)

Therefore, the popularity of AI in China is relatively high, and the respondents had a certain understanding of artificial intelligence (such as alpha go, etc.), but there were still deficiencies in professional fields (such as ophthalmology). The most of respondents had a high degree of acceptance of AI in ophthalmology, and held a positive attitude towards its current development, believing that AI can improve the quality of people’s lives. Although most respondents had no experience in ophthalmic AI, they still believed that ophthalmic AI would partially replace doctors. But doctors played a vital role in the diagnosis and treatment of diseases and would not be completely replaced.

### Comparison of perceptions and attitudes towards AI between medical workers and other professional technicians

**Fig. 1:**
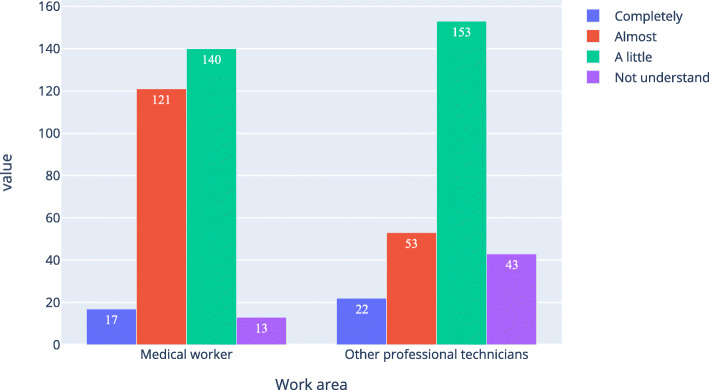
Respondents’ understanding level of artificial intelligence

**Fig. 2:**
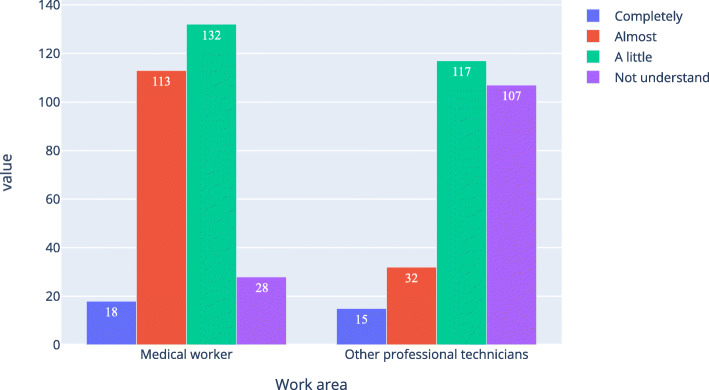
Respondents’ understanding level of medical artificial intelligence

**Fig. 3:**
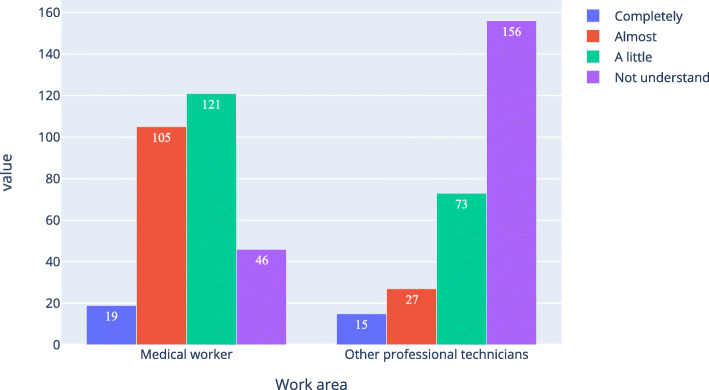
Respondents’ understanding level of ophthalmic artificial intelligence

Table 4; Figs. 1 and 2, and Fig. 3 show that the proportion of medical workers whose understanding level was “completely understand” or “almost understand” was 47.4 %, 45.0 %, and 42.6 %, respectively, for AI, medical AI, and AI in ophthalmology. For other professional technicians, the proportion was 27.7 %, 17.4 %, and 15.5 %, respectively. The Pearson Chi-Square of work area and understanding level for AI, medical AI, and AI in ophthalmology was 43.207, 92.059, and 117.776 respectively. All the P was 0, which means the work area of the respondents was related to their understanding of AI, medical AI, and AI in ophthalmology. Table 5 was a simple version of Table 4. In Table 5, option “completely” and “almost” were merged together, option “a little” and “not understand” were merged together. As shown in Table 4, all the P were equal 0, all the odds ratio (OR) were bigger than 1 and all the lower bound of the 95 %CI were bigger than 1.

**Table 4:** Medical workers and other professional technicians ' basic understanding of artificial intelligence (N of medical workers = 291, N of other professional technicians = 271)

Understanding level	Medical workers n(%)	Other professional technicians,n(%)	Chi-Square,X^2^	Asymp. Sig., P
**Artificial intelligence**				
Completely	17(5.8)	22(8.1)	43.207	0
Almost	121(41.6)	53(19.6)		
A little	140(48.1)	153(56.5)		
Not understand	13(4.5)	43(15.9)		
**Medical artificial intelligence**				
Completely	18(6.2)	15(5.5)	92.059	0
Almost	113(38.8)	32(11.8)		
A little	132(45.4)	117(43.2)		
Not understand	28(9.642)	107(39.5)		
**Ophthalmic artificial intelligence**				
Completely	19(6.543)	15(5.5)	117.776	0
Almost	105(36.1)	27(10.0)		
A little	121(41.6)	73(26.9)		
Not understand	46(15.8)	156(57.6)

**Table 5:** Simple version of medical workers and other professional technicians ' basic understanding of artificial intelligence (N of medical workers = 291, N of other professional technicians = 271)

Understanding level	Medical workers, n(%)	other professional technicians, n(%)	Chi-Square, X2	Asymp. Sig., P	OR	95 %CI
**Artificial intelligence**						
Completely and Almost	138(47.4)	75(27.7)	23.249	0	2.357	1.658–3.351
A little and Not understand	153(52.6)	196(72.3)				
**Medical artificial intelligence**						
Completely and Almost	131(45.0)	47(17.4)	49.658	0	3.902	2.642–5.764
A little and Not understand	160(55.0)	224(82.7)				
**Ophthalmic artificial intelligence**						
Completely and Almost	124(42.6)	42(15.5)	49.564	0	4.048	2.706–6.056
A little and Not understand	167(57.4)	229(84.5)				

**Fig. 4:**
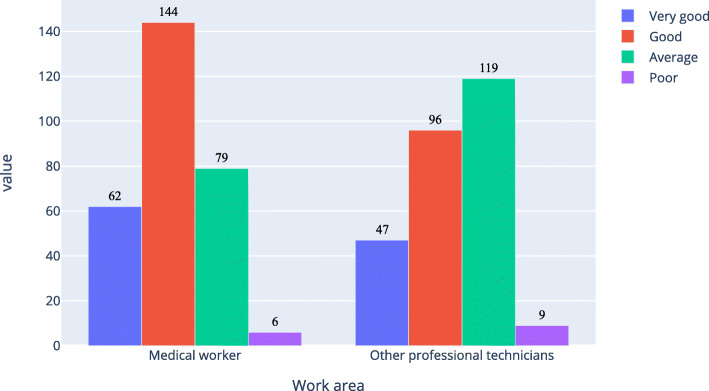
Respondents’ evaluation of the current development level of artificial intelligence

**Fig. 5:**
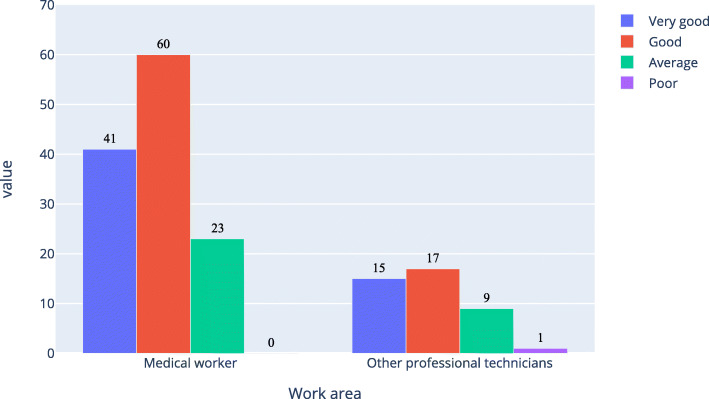
Evaluation of the current development level of artificial intelligence of the respondents who completely and almost understood artificial intelligence

As shown in Figs. 4 and 5, about 71 % of medical workers and 53 % of other professional technicians believed that the current development of AI in ophthalmology was good; while among those who completely understood and almost understood AI in ophthalmology, about 81 % of medical workers and 76 % of other professional technicians thought that the current development of AI in ophthalmology was good.

For the two different groups by working fields, the respondents’ understanding level of AI, medical AI, and AI in ophthalmology tended to drop in the mentioned order. The proportion of respondents whose understanding level of these three AIs was “completely understand” or “almost understand” was greater among medical workers than among other professional technicians. The close combination of medicine and AI had enabled medical workers to understand more about AI. Therefore, AI is relatively well popularized in the medical field; meanwhile, there is a need to enhance the popularization of artificial intelligence-related knowledge among people in other fields.

**Fig. 6:**
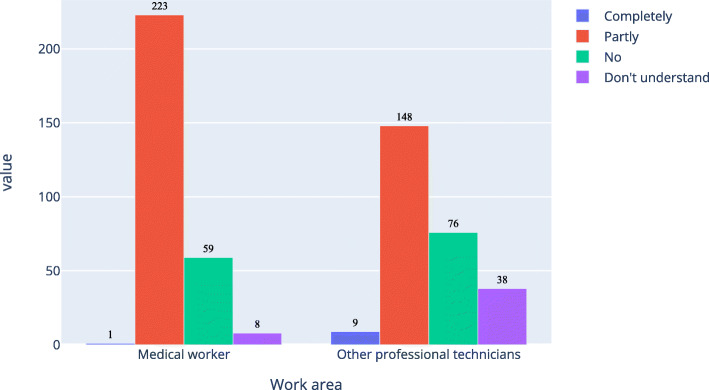
Respondents’ prediction on the extent to which ophthalmic artificial intelligence would replace doctors

**Fig. 7:**
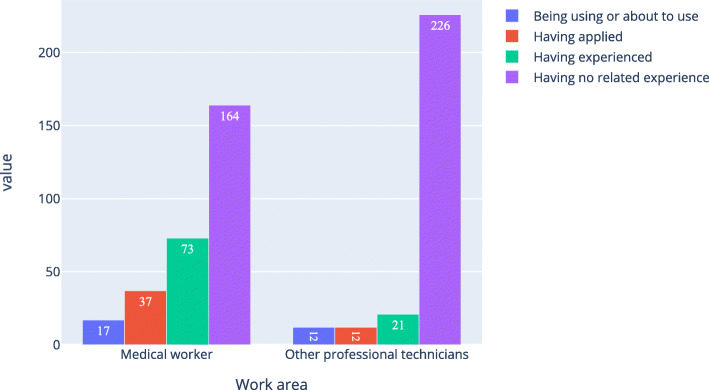
Respondents’ experience in the application of ophthalmic artificial intelligence

**Fig. 8:**
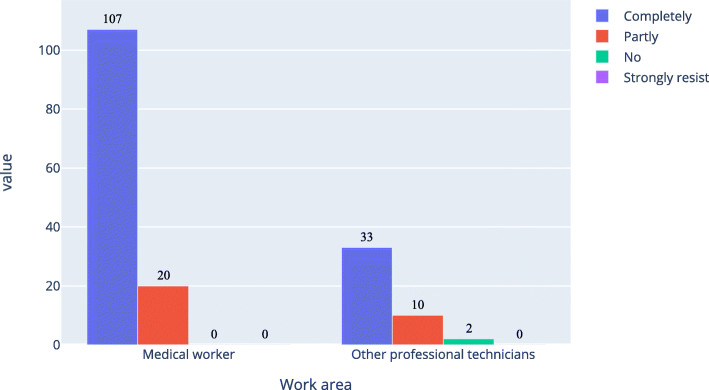
Acceptance level of ophthalmic artificial intelligence among the respondents who had experience in ophthalmic artificial intelligence

As shown in Figs. 6, 7 and 8, about 77.0 % of medical workers and 57.9 % of other professional technicians believed that AI in ophthalmology would completely or partly replace doctors. There were 56.4 % of medical workers and 83.4 % of other professional technicians had no experience in the application of AI in ophthalmology, while among those with experience in the application of AI in ophthalmology, there were 84.3 % of medical workers and 73.3 % of other professional technicians would fully accept it.

x

In Table 6, option “completely” and “partly” were merged together, option “no” and “don’t understand” were merged together, option “no” and “strongly resist” were merged together; for question “Do you have an experience with an ophthalmic artificial intelligence application?”, option “having no related experience” was divided into one group, other options were combined into another group. As shown in Table 6, the P of the first and second questions were equal 0, the odds ratio (OR) of them were bigger than 1 and the lower bound of the 95 %CI of them were bigger than 1. So the work area of the respondents was related to the two questions. The P of the third question was 0.061, the OR was 2.357 but the 95 %CI was 0.883–12.312. So it wasn’t related to the work area of the respondents.

**Table 6:** Simple version of medical workers and other professional technicians ' attitudes of artificial intelligence (N of medical workers = 291, N of other professional technicians = 271)

Attitude	Medical workers, n	other professional technicians, n	Chi-Square, X2	Asymp. Sig., P	OR	95 %CI
**AI in ophthalmology will replace ophthalmologists**						
Completely and Partly	224	157	23.304	0	2.428	1.687–3.494
Not and Don’t understand	67	114				
**Do you have any experience with AI in ophthalmology?**						
Being using or about to use, Having applied and Having experienced	127	45	48.299	0	3.889	2.620–5.773
Having no related experience	164	226				
**Acceptance level of AI in ophthalmology**						
Completely and Partly	288	262	3.522	0.061	2.357	0.883–12.312
Not accept and Strongly resist	3	9				

For the two groups by working fields, most of people did not have experience in applying artificial intelligence to ophthalmology, but still believe that AI will partially replace doctors, which shows that the respondents have a certain understanding of AI, but AI had not been widely used in ophthalmology. Compared with other professional technicians, medical workers had more experience in AI. AI was developing rapidly in ophthalmology and had better prospects. There was only 1.8 % of the respondents believed that AI would completely replace ophthalmologists, indicating that people recognized the value of ophthalmologists and believed that even if AI developed better in the future, it is still only an aid to ophthalmologists rather than a replacement. At the same time, there was only 2.1 % of the respondents had an unacceptable attitude towards AI in ophthalmology, indicating that people recognized the combination of AI and the ophthalmology. This recognition had nothing to do with the working fields, but people believed that AI could improve people’s lives.

### Respondents’ concerns about artificial intelligence

**Fig. 9:**
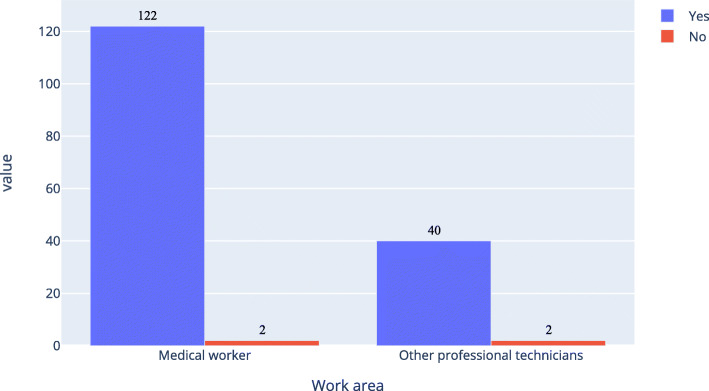
Attitudes of the respondents whose understanding level was “almost understand” or above toward the question

In Table 7, among the respondents, 56.4 % said that in the current ophthalmic AI practice, medical responsibilities are unclear; 49.3 % said that the quality of AI in ophthalmology services was difficult to guarantee; while the percentage of those who thought there existed problems such as extreme high prices, medical ethical risks and lack of political support was about 40 %. More than 90 % of the respondents thought there was a need to strengthen medical ethics research in the ophthalmic AI field. Among those who completely and almost understood AI in ophthalmology, 98.4 % of medical workers and 95.2 % of other professional technicians believed it was necessary to strengthen medical ethics research in the field, as shown in Fig. 9. This is enough to showcase the importance of addressing medical ethical issues in the ophthalmic AI field.

**Table.7:** Respondents’ concerns about artificial intelligence (*N* = 562)

What are your concerns about AI in ophthalmology? (multiple selection)	Respondents n(%)
Medical responsibilities are unclear	317(56.4)
Service price is too expensive	252(44.8)
Service quality is difficult to guarantee	277(49.3)
Medical ethical risk	240(42.7)
Policy support may not be in place	235(41.8)
Others	85(15.1)
**Is it necessary to strengthen the study of medical ethics in the ophthalmic AI field?**	Respondents n**(%)**
Yes	517(92.0)
No	45(8.0)

## Discussion

Among the medical workers, there was 42.6 % could understand ophthalmic AI, about 84.3 % fully accepted ophthalmic AI, there was 43.6 % had related applications experience in ophthalmic AI, there was 77 % believed that ophthalmic AI would completely or partially replace doctors, and there was 98.4 % believed that ophthalmic AI ethics research needs to be strengthened. These results help to make some targeted improvements in ophthalmic AI research, such as setting up more pilots so that people have more opportunities to experience ophthalmic AI and more accept ophthalmic AI, therefore, the application of ophthalmic AI can be promoted to the clinic. During diagnosis and treatment, the workload of medical workers is reduced.

In the survey, the proportion of medical workers was roughly equal to the proportion of other professional technicians. The proportion of respondents who did not understand AI among other professional technicians was about three times that of medical workers. And the proportion of those who didn’t understand medical AI among other professional technicians was about 4 times that of medical workers. The proportion of people who did not understand AI in ophthalmology among other professional technicians was about four times that of medical workers. Hence one can see that the understanding level of AI, medical AI, and ophthalmic AI among the medical workers who participated in the survey is higher than that among other professional technicians, suggesting that in China, AI is relatively more widely popularized among medical workers. In recent years, AI has been more and more widely introduced in the medical field [16, 31–34]. Due to work or academic exchanges, medical workers have more opportunities to learn about medical AI than other professional technicians. The other professional technicians in this survey were technical personnel whose research areas had no connection to medicine or AI. As a result, they had relatively less knowledge about medical AI and fewer opportunities to apply or experience medical AI, especially in the more specialized clinical ophthalmology field. Naturally, those in the survey who had no medical background would have a difficult time imagining the application of AI in medicine. Therefore, the real-life application of AI in other areas should be further popularized in our society to gain more of people’s trust before AI can be used in medical care.

Concerning the attitudes toward AI, both medical workers and other professional technicians were relatively confident in human doctors, with only a very small proportion of people thinking that AI in ophthalmology would completely replace ophthalmologists. Most respondents were relatively rational, believing that ophthalmic AI would only partly replace ophthalmologists. As people become more rational about AI in ophthalmology, the condition has been more and more favorable for the healthy development of AI in the medical field. As suggested by Turing for the healthy development of AI, “Instead of trying to produce a program to simulate the adult mind, why not rather try to produce one which simulates the child’s? If this were then subjected to an appropriate course of education one would obtain the adult brain.” [35] This conception has functioned as guidance in the research of medical AI. From JAMA’s publication of AI used for DR diagnosis in 2016 [16] to the U.S. Food and Drug Administration’s approval of IDx-DR in 2018 [25], there has been no real diagnostic systems that can fully diagnose and identify all the 4 grades of DR, which means there is still a long way for ophthalmic AI to go from laboratory research to clinical application. But undeniably, in a country such as China where ophthalmologists are in much dire need (the over 1.4 billion Chinese people only share about 44,800 ophthalmologists) [36], the application of AI can help to diagnose and treat many more patients, which would reduce the burden on ophthalmologists, thus probably having led to the higher acceptance level of ophthalmic AI among medical workers. Meanwhile, those other professional technicians, despite a relatively lower understanding level, also basically held a positive attitude to acknowledging AI in ophthalmology.

As far as the application of ophthalmic AI goes, there are currently some pilot centers in China that provide opportunities to experience its real-life application. However, due to the rarity of such centers, only close to half of the medical workers involved in the survey had related experience while 4/5 of the other professional technicians had no such experience. This indicates that the application of AI in ophthalmology is not yet popularized. It has very few real-life applications, mainly due to technical and ethical issues. On the technical side, the systems that had relatively good diagnosing abilities turned out to be not well performed in the complex real-life scenarios, requiring further improvement. And on the ethics side, the medical responsibilities are not clearly defined for the artificial intelligence diagnostic systems and there are no related policies to follow or to regulate it with. Therefore, although the research of AI in ophthalmology is going well, more pilot centers are needed to explore the problems that may be encountered before actual application; it still takes prudence in its real-life application.

In recent years, the application of AI in ophthalmology is very deeply researched, but there are not so many studies on related policies and ethics. The survey found that unclear medical responsibilities and difficulty in guaranteeing service quality respectively ranked as the No. 1 and No. 2 concerns about the use of AI in ophthalmology, with 60 % of the respondents worrying about the “unclear medical responsibilities.” These concerns are sufficient to show that the country needs to improve its regulation system of AI and strengthen the exploration of relevant medical ethics issues. Only when the relevant regulation system and ethics issues have been addressed can we guarantee the real-life practice of medical AI, and establish people’s confidence in medical AI so that they can truly accept its relevant application. Therefore, the next step is to research on AI technology that can eliminate ethical risks and non-technical countermeasures, etc.

The survey mainly analyzed the respondents’ understanding and acceptance level of AI in ophthalmology, as well as the respondents’ concerns about AI in ophthalmology. The respondents in this survey are mainly from Zhejiang, China, an important part of the Yangtze River Delta, which is one of china’s relatively developed regions. With almost no respondents from remote areas, the survey results do not represent those of medical workers and other professional technicians nationwide. Besides, although the respondents are relatively evenly distributed among the different groups, the total number is relatively small. The subjects of the survey are mainly divided into medical workers and other professional technicians. This survey mainly studies the understanding and attitudes of ophthalmology AI in different working fields, and the research angle is relatively single. The follow-up surveys should try to broaden the survey scope and increase the angle of research angle (doctors, patients, etc.), making the findings more credible and broadly representative.

## Conclusions

The survey results revealed that the medical workers had a higher understanding level of AI in ophthalmology than other professional technicians, making it necessary to popularize ophthalmic AI education among other professional technicians. Most of the respondents did not have any experience in ophthalmic AI, but generally had a relatively high acceptance level of AI in ophthalmology, and there was a need to strengthen research into the medical ethics issues of the field. The next step of this research is to expand the scope of survey, increase the angle of survey, and carry out research on AI technology that can eliminate ethical risks and research on non-technical countermeasures,promoting the clinical application of AI.

## Data Availability

The data and materials used and/or analyzed during the present study are available from the corresponding author on reasonable request.

## Abbreviations

AI: Artificial intelligence
OCT: Optical coherence tomography
DR: Diabetic retinopathy
